# The concurrence of lung malignancies and organizing pneumonia

**DOI:** 10.3389/fonc.2025.1733651

**Published:** 2026-01-16

**Authors:** Srikanth Vedachalam, Mohammad Freihat, Ahmed K. Alomari, Catherine R. Sears, Nawar Al Nasrallah

**Affiliations:** 1 Division of Pulmonary, Critical Care & Sleep Medicine, University of Cincinnati College of Medicine, Cincinnati, OH, United States; 2 Department of Medicine, Indiana University School of Medicine, Indianapolis, IN, United States; 3 Division of Clinical Pathology and Laboratory Medicine, Indiana University School of Medicine, Indianapolis, IN, United States; 4 Division of Pulmonary, Critical Care, Sleep and Occupational Medicine, Indiana University School of Medicine, Indianapolis, IN, United States; 5 Richard L. Roudebush Veterans Affairs Medical Center, Indianapolis, IN, United States

**Keywords:** lung adenocarcinoma, lung cancer, organizing pneumonia, squamous cell carcinoma, transbronchial lung biopsy

## Abstract

**Introduction:**

Organizing pneumonia (OP) is often considered a benign finding, but in some cases, it may be associated with underlying malignancy. With the increasing use of minimally invasive biopsy techniques, understanding the relationship between OP and lung cancer is critical to avoid delayed or missed diagnoses. This study aims to evaluate the prevalence of OP and lung malignancy and report characteristics of these cases.

**Material and methods:**

A retrospective review was conducted of all lung pathology reports at Indiana University from January 1, 2010, to January 1, 2023, encompassing over 8,000 cases. Cases with histopathologic diagnoses of OP with and without lung malignancy were identified; relevant clinical, radiologic, and follow-up data were extracted. Using language extraction, we identified pathology cases showing both organizing pneumonia and carcinoma (Cohort 1) and transbronchial biopsies identified as “organizing pneumonia” (Cohort 2). Cohort 2 was selected to reflect current clinical practice.

**Results:**

In Cohort 1 (n=57), 88% of samples demonstrating both organizing pneumonia and malignancy were obtained via surgical resection. Among these, squamous cell carcinoma accounted for 46% and adenocarcinoma for 37% of cases, with all T-stages represented (44% T1, 29% T2, 16% T3, and 11% T4). In this cohort, thirty-eight prior biopsy samples prior to surgery were reviewed; 76% revealed malignancy, but none showed definitive organizing pneumonia likely due to focal nature of OP. Cohort 2 (n=40), 7.5% of patients in whom organizing pneumonia was identified through minimally invasive bronchoscopic sampling were ultimately diagnosed with concurrent lung cancer. All required repeat tissue sampling for cancer diagnosis.

**Conclusion:**

Lung malignancy is known to be associated with OP. A high degree of suspicion should be maintained if OP is found on minimally invasive techniques, particularly if malignancy is strongly suspected.

## Introduction

Lung cancer remains as the primary cause of cancer-related mortality in both the United States and globally (1).Early detection of lung cancer significantly increases the chances of successful treatment and cure. Early stage lung cancer, presenting as a pulmonary nodule, can be difficult to distinguish from benign causes. An increase in pulmonary nodules has corresponded to increased use of computed tomography (CT) chest imaging in clinical care and lung cancer screening (2). This has paralleled increased utilization of percutaneous and bronchoscopic biopsies for lung cancer diagnosis (3, 4). Recent guidelines highlight the importance of differentiating specific malignant and benign pathologic diagnoses from non-diagnostic, non-specific findings (5). This is critical to maximize diagnostic accuracy and ultimately to ensure timely care for patients undergoing bronchoscopic procedures (6).

One of these benign pathologic diagnoses is organizing pneumonia (OP). OP can be classified as primary, also known as a cryptogenic organizing pneumonia, and secondary OP, the latter of which forms the larger group of OP seen in clinical practice (76.2%) (7). Further complicating the matter is the variety of conditions associated with secondary OP, which include infections, connective tissue diseases, cancer-based treatments, environmental exposures, organ transplantations, and also occult malignancy (8–11). There have been case reports where OP may be the only sign of malignancy and may not necessarily be adjacent to the primary tumor (12–14). In several instances, malignancy was missed for years despite multiple minimally invasive procedures, such as transbronchial lung biopsies (TBLB), CT-guided biopsies, and lymph node sampling. These findings highlight the need for better characterization of malignancy-associated OP and a deeper understanding of its clinical significance when identified on lung biopsy.

The aim of this study is to assess the prevalence of organizing pneumonia (OP) in association with lung cancer. We hypothesize that OP is not uncommonly identified concurrently with pulmonary malignancy. This retrospective analysis evaluates the frequency of combined pathologic findings in tissue samples, determines the prevalence of lung cancer in bronchoscopically obtained transbronchial biopsies classified as OP, and describes associated tumor and patient characteristics. Understanding the risk of concurrent lung cancer in patients diagnosed with OP is critical to avoid delayed cancer diagnoses and to prevent the misclassification of a potentially malignant process as benign.

## Materials and methods

### Patient selection

This single-center retrospective study was conducted at Indiana University Health, a tertiary academic health center. The study was granted exemption from full institutional review board (IRB) review (IRB #18034) by the Indiana University School of Medicine (IUSM). To identify relevant cases, we performed two keyword-based searches of the IUSM patient database for pathology reports dated between January 1, 2010, and January 1, 2023, which included more than eight thousand lung pathology reports. Diagnoses, histologic and de-identified demographic data were extracted for descriptive analyses via electronic medical record (EMR) chart review. Personally identifiable information was accessible only to authorized research personnel and securely stored in password-protected files.

The first search (Cohort 1) targeted reports containing the terms “organizing,” “pneumonia,” and “carcinoma,” and yielded 70 patient records. Inclusion criteria required adult patients to have documented diagnoses of both malignancy and organizing pneumonia (OP). Following a quality assurance review of patient charts, 13 individuals were excluded for not meeting both criteria, resulting in a final cohort of 57 patients.

The second search (Cohort 2) used the term “organizing pneumonia” and identified over 350 cases during the study period. From this group, we included only patients who underwent transbronchial lung biopsy (TBLB) of native lung tissue to avoid overlap with the initial cohort, which primarily consisted of surgical lung biopsies, resections, and lung transplant recipients—the latter representing the majority of OP diagnoses in that group. After applying these criteria, 40 patients diagnosed with OP by TBLB were included in the final cohort. We retrospectively followed these patients for at least two years using the EMR to assess outcomes, including repeat lung biopsies and subsequent diagnoses of malignancy.

### Data collection and outcomes

Patient data were manually abstracted from electronic medical records. Demographic variables included age, gender, and race. Specimen characteristics were documented, including specimen type (surgical resection, core biopsy, transbronchial biopsy, fine needle aspirate, and other), final pathology classification (adenocarcinoma, squamous cell carcinoma, small cell carcinoma, other primary lung malignancies, and secondary carcinomas), and the anatomical relationship of organizing pneumonia (OP) to the carcinoma (adjacent to the tumor, within the same lobe but not directly involved, or located in other lobe[s]). Tumor size was recorded from both pathology reports and CT imaging (in centimeters), along with surgical T-staging based on the eighth edition of the tumor, node, metastasis (TNM) staging system. Prior biopsy attempts were noted by type and categorized by outcome: malignancy, inflammation/necrosis, OP, granuloma, or non-diagnostic.

### Statistical consideration

Redcap was utilized for all data collection. Demographics, final pathology, tumor stage and location of organizing pneumonia in comparison to lung cancer are presented as percentages and absolute counts. Tumor size on pathology reports and on CT scans were reported as mean values with associated ranges. Categorical CT features were compared using Fisher’s exact test for binary variables and permutation-based chi-square for multi-category variables. Odds ratios with 95% confidence intervals were calculated with continuity correction for zero cells. A p-value <0.05 was considered significant.

## Results

### Demographics

#### Cohort 1: Tumor and organizing pneumonia characteristics

First, we examined the association between lung cancer and OP in Cohort 1. Cohort 1 included a total of 57 patients, as detailed in **Table 1**. The median age was 67 years (mean = 66; range: 36–86 years). Females comprised 51% of the cohort. In terms of self-reported race, 67% were White, 5% Black, 2% Asian, and 26% identified as “Other.” Pathologic specimen types were predominantly surgical resection samples, comprising 88% (n = 50) of the total cohort. Core biopsy samples accounted for 4% (n = 2), transbronchial biopsies 5% (n = 3), fine needle aspirates 4% (n = 2), and “other” biopsies (obtained during rigid bronchoscopy) 2% (n = 1). Final pathology was predominantly composed of non-small cell lung cancer subtypes: 46% of patients had squamous cell carcinoma, 37% had adenocarcinoma, 9% had “other” lung primaries, and 12% had secondary non-lung primary carcinomas. None of the samples showed small cell lung cancer.

**Table 1:** Demographics and tumor characteristics of cohort 1.

Participants	
N	57
Gender - % (n)	
Female	51% (29)
Race- % (n)	
Black	5% (3)
White	67% (38)
Asian	2% (1)
Other	26% (15)
Pathologic Specimen Type- % (n)	
Surgical	88% (50)
Core Biopsy	4% (2)
Transbronchial Biopsy	5% (3)
Fine Needle Aspirate	4% (2)
Other	2% (1)
Final Pathology- % (n)	
Adenocarcinoma	37% (21)
Squamous Cell Carcinoma	46% (26)
Small Cell	0% (0)
Other Lung Primary	9% (5)
Metastatic Carcinoma	12% (7)
Location of Groundglass Opacity to Carcinoma- % (n)	
Same Lobe, Adjacent Lung Tissue	68% (39)
Same Lobe, Distinct Lung Tissue	14% (8)
Different Lobes	28% (16)
Tumor Size (Pathology) (cm) Mean (Range)	3.61 (0.3-11.4)
Tumor Size (CT) (cm) Mean (Range)	3.56 (0.8-13.9)
Primary Tumor T-Staging (n=45)- % (n)	
T1	44% (20)
T2	29% (13)
T3	16% (7)
T4	11% (5)

Values are presented as percentages, with absolute numbers shown in parentheses.

**Figure 1:**
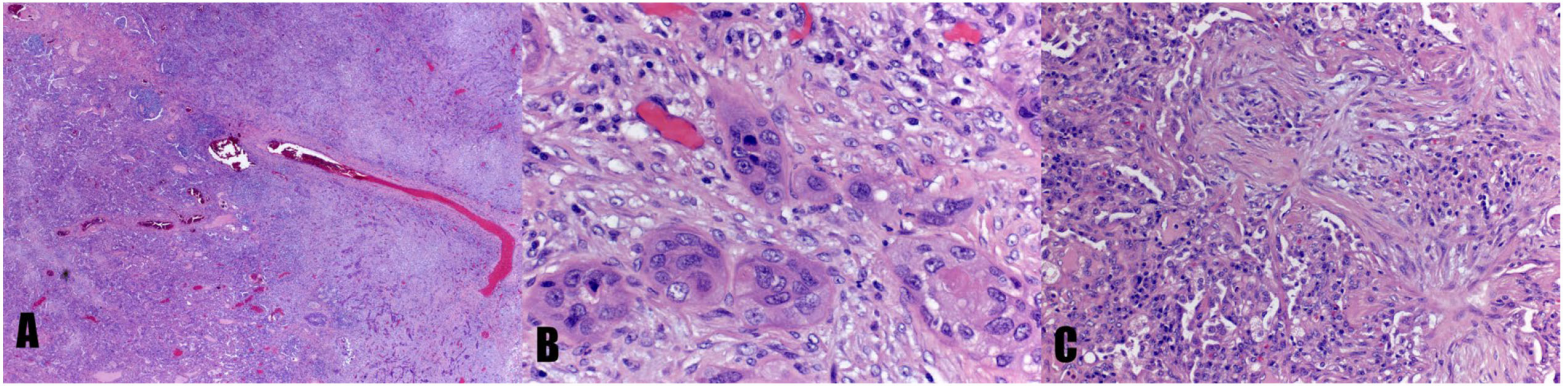
Histology of coexisting squamous cell carcinoma and organizing pneumonia in the same lung specimen. **(A)** Squamous cell carcinoma of the lung (right half) and organizing pneumonia (left half) in a low power field (40x). **(B)** Squamous cell carcinoma of the lung in a high power field (400x). **(C)** Organizing pneumonia in a high power field (200x).

Detailed data regarding tumor and OP characteristics are presented in **Table 1**. The mean tumor size was 3.61 cm as reported by pathology (range: 0.3–11.4 cm), and 3.56 cm as reported on CT chest imaging (range: 0.8–13.9 cm). Tumor T-stage was categorized as follows: 44% were T1, 29% T2, 16% T3, and 11% T4. The location of OP in relation to malignancy was most often adjacent to the tumor, found in 68% of cases (**Figure 1**). Additionally, 14% of cases had OP in the same lobe but not adjacent to the tumor, and 28% had OP diagnosed in a separate lobe. These later cases were typically observed in specimens from pneumonectomies.

We also identified prior biopsy attempts in patients from Cohort 1 with confirmed malignancy and organizing pneumonia (OP). Out of 57 patients, 34 had documented biopsies in our electronic medical record system (**Table 2**). The remaining patients were either referred directly for surgery due to high clinical suspicion or underwent diagnostic procedures at external facilities, with results unavailable for review. Four patients had two prior biopsy attempts on record. Of the 38 total biopsies analyzed, three procedures involved two distinct biopsy techniques, and one procedure involved three. Among patients who underwent surgical resection, the average interval between the preceding biopsy and the resection procedure was approximately 33 days.

**Table 2:** Sampling techniques in pre-surgical procedures for cohort 1 (N = 38).

Type of prior biopsies/sampling- % (n)	
Fine Needle Aspiration	34% (13)
Transbronchial Lung Biopsy	21% (8)
Endobronchial Biopsy	18% (7)
Brush Biopsy	3% (1)
Fluid Cytology	13% (5)
CT Guided Core Needle Biopsy	24% (9)
Final Pathology on Prior Biopsies- % (n)	
Malignancy	76% (29)
Inflammation/Necrosis	13% (5)
Organizing Pneumonia	0% (0)
Granuloma	0% (0)
Non-Diagnostic	16% (6)
Biopsy-to-Resection Time	
Average Time from Biopsy to Resection (Days)	32.8

Fine needle aspiration was used in 34% of procedures, transbronchial biopsy in 21%, endobronchial biopsy in 18%, brushings in 3%, bronchoalveolar lavage fluid cytology in 13%, and CT-guided core needle biopsy in 24%. Among these, 76% revealed malignancy, 13% showed inflammation or necrosis, and 16% were non-diagnostic. Importantly, none of the biopsies clearly mentioned organizing pneumonia, even though it included inflammation (**Table 2**).

#### Cohort 2: Diagnostic implications of OP on TBLB: assessing the risk of missed malignancy

As previously noted, none of the lung biopsies in Cohort 1 demonstrated organizing pneumonia (OP) prior to surgical resection. To evaluate whether the presence of OP on transbronchial lung biopsy (TBLB) reduces the likelihood of concurrent carcinoma—or whether this reflects selection or sampling bias in Cohort 1, we analyzed Cohort 2 in which OP was diagnosed via TBLB. Cohort 2 consisted of 40 patients, with a median age of 63 years (mean = 58.5; range: 18–86 years). The majority were male (63%) and White (92.5%). Among these cases of OP diagnosed by TBLB, only one case showed carcinoma concurrently in the initial TBLB pathology report. Four additional patients (10%) were later diagnosed with malignancy in the same anatomical region during follow-up, based on repeat lung biopsies or surgical resection (**Table 3**). One case was identified as a neuroendocrine tumor after evaluation of multisite involvement. These findings highlight that a diagnosis of OP on transbronchial lung biopsy (TBLB) may not reliably exclude underlying malignancy, underscoring the need for continued clinical vigilance and follow-up when suspicion persists.

**Table 3:** Demographics and tumor characteristics of cohort 2.

Participants	
N	40
Gender - % (n)	
Male	63% (25)
Race- % (n)	
Black	7.5% (3)
White	92.5% (37)
Pathology- % (n)	
OP alone	47% (19)
OP and Carcinoma	2.5% (1)
OP and Granuloma	17.5% (7)
OP and other inflammatory changes/fibrosis	25% (10)
OP and fungal infection	7.5% (3)
Second biopsy if applicable	
Open lung biopsy	10% (4)
CT-guided biopsy	2.5% (1)
TBLB	2.5% (1)
Follow-up diagnosis of malignancy	10% (4)
Lung Adenocarcinoma	2.5% (1)
Neuroendocrine tumor	2.5% (1)
High grade sarcoma	2.5% (1)
Renal Cell Carcinoma	2.5% (1)

OP, organizing pneumonia, TBLB, Transbronchial lung biopsy.

#### CT imaging features in carcinoma-associated OP vs other OP

**Figure 2:**
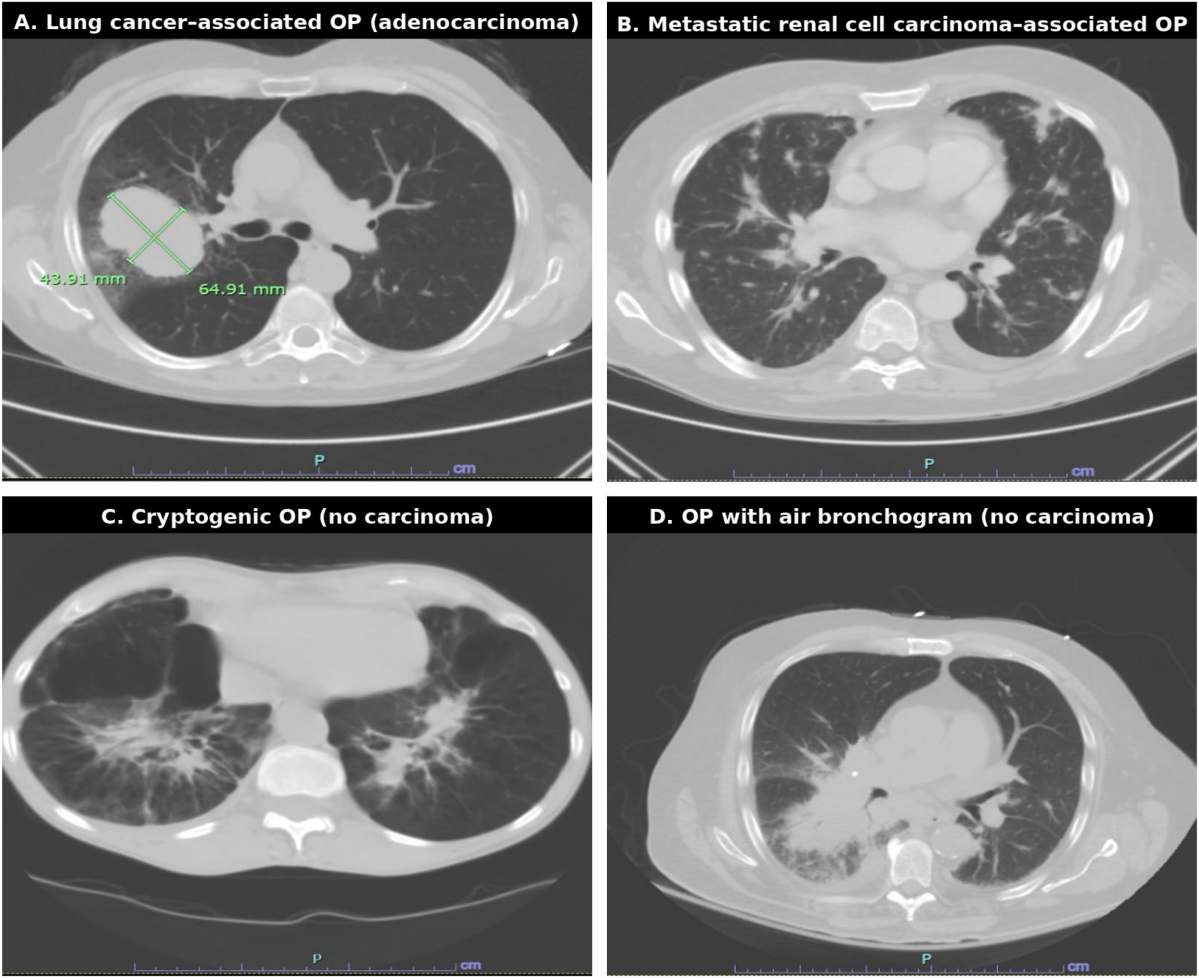
Organizing pneumonia subtypes on CT. Representative CT images of organizing pneumonia (OP): **(A)** Lung cancer–associated OP (adenocarcinoma). **(B)** OP associated with metastatic renal cell carcinoma. **(C)** Cryptogenic OP (no carcinoma). **(D)** OP with air bronchogram (no carcinoma).

Among 39 patients (5 with carcinoma-associated OP and 34 with other OP), CT scans were available for review by the study team for all cases but one case in Cohort 2. As shown in **Table 4**, CT features demonstrated no significant differences in lesion morphology (p=0.477), lobar involvement (p=0.634), margins (p=1.000), location (p=0.857), or air bronchogram (p=0.563). Pulmonary mass was more frequent in carcinoma-associated OP (40% vs 17.6%), while cavitary lesions predominated in other OP (47.1% vs 20%), but these trends were not statistically significant. Air bronchogram was absent in all carcinoma-associated OP cases yet present in 23.5% of other OP cases; however, Fisher’s exact test remained non-significant due to small sample size and wide confidence intervals (OR 0.28, 95% CI 0.01–5.67). The only significant finding was thoracic lymphadenopathy, observed in 100% of carcinoma-associated OP versus 40% of other OP (p=0.0177), suggesting its potential role as an imaging marker for underlying malignancy. **Figure 2** shows different examples of CT findings in the setting of cryptogenic OP, OP and carcinoma associated OP.

**Table 4:** CT imaging features: carcinoma-associated OP vs other OP.

Feature	Category	Carcinoma-associated OP (count, %)	Other OP (count, %)	P-value
Lesion morphology (overall)				0.477
	Solitary pulmonary nodule (SPN)	0 (0.0%)	1 (2.9%)	1.0000
	Multiple pulmonary nodules	1 (20.0%)	9 (26.5%)	1.0000
	Pulmonary mass	2 (40.0%)	6 (17.6%)	0.2677
	Cavitary pulmonary lesion	1 (20.0%)	16 (47.1%)	0.3634
	Pulmonary infiltrate	1 (20.0%)	2 (5.9%)	0.3452
Lobar involvement				0.634
	Single lobe	3 (60.0%)	14 (40.0%)	
	More than one lobe	2 (40.0%)	21 (60.0%)	
Lesion margins				1.000
	Spiculated	5 (100.0%)	34 (97.1%)	
	Smooth	0 (0.0%)	1 (2.9%)	
Lesion location (overall)				0.857
	Central	1 (20.0%)	4 (11.8%)	0.5167
	Peripheral	3 (60.0%)	18 (52.9%)	1.0000
	Both	1 (20.0%)	12 (35.3%)	0.6478
Lymphadenopathy				**0.0177**
	Present	5 (100.0%)	14 (40.0%)	
	Absent	0 (0.0%)	21 (60.0%)	
Air bronchogram				0.563
	Present	0 (0.0%)	8 (23.5%)	
	Absent	5 (100.0%)	26 (76.5%)	

Values shown in bold indicate a p-value less than 0.05, which is considered statistically significant.

## Discussion

This study presents one of the largest case series of Organizing pneumonia (OP) and concurrent lung cancer, helping to further characterize this phenomenon. Correlation between lung malignancy and OP has been described and may be the only sign of malignancy in some cases (15). OP is a relatively common concurrent finding in lung cancer, observed in up to 37% of resected specimens. However, the mechanisms underlying this association remain poorly understood (15). Proposed explanations include bronchial obstruction from malignancy leading to OP, chronic inflammation from OP increasing cancer risk, and OP triggered by prior chemotherapy or radiation (7, 12–14, 16). Our study examined a total of 61 cases of malignancy associated with OP over a 13-year period. The predominant malignancies associated with OP were non-small cell subtypes, particularly squamous cell carcinoma and adenocarcinoma. Most specimens were obtained via surgical resection rather than minimally invasive techniques. Only one minimally invasive biopsy showed malignancy and OP concurrently. Additionally, our study found that OP can be associated with Lung malignancies of all T-stages. While lower stages were more common (44% T1 and 29% T2), a significant proportion of higher-stage tumors was also observed (16% T3 and 11% T4).

Squamous cell carcinoma accounted for 46% of cases which is higher than the expected 25–30% in the general NSCLC population, whereas adenocarcinoma typically comprises about 40% and large cell carcinoma 10–15% (17, 18). This finding is consistent with previous reports by Romero et al., suggesting a potential association between OP and lung squamous cell carcinoma (15). One hypothesis is that the typical central location of squamous tumors may predispose to airway obstruction and a subsequent inflammatory response, which can trigger OP; however, this remains speculative and requires further investigation (19).

Multiple studies have noted that cancer diagnoses can be missed with less invasive techniques, as surgical specimens may reveal scattered tumor cells within a background of OP (12, 14, 15, 20). In our study, 88% of Cohort 1 patients underwent surgical lung biopsy or resection, and 38 of 50 had prior bronchoscopic or CT-guided biopsies. None of these initial biopsies demonstrated histologic evidence of organizing pneumonia (OP), although tissue or cytology was often described as inflammatory, non-diagnostic, or suspicious. Despite prior biopsies, malignancy was ultimately confirmed in 76% of cases, prompting surgical resection. This discrepancy likely reflects sampling limitations with fine needle aspiration or small transbronchial biopsies and the patchy nature of OP, which can be missed in limited samples (19). To further explore this issue, we examined a second cohort of 40 patients diagnosed with OP via transbronchial lung biopsy (TBLB) and subsequently followed for at least two years. While only one patient had carcinoma identified at the time of initial biopsy, four additional patients (10%) were later diagnosed with malignancy from the same anatomical region through repeat biopsy or surgical resection. These findings are consistent with prior reports, including one study where 11.2% of patients with OP diagnosed on CT-guided biopsy were ultimately found to have malignancy (20). This highlights an important clinical consideration: a diagnosis of OP does not exclude other etiologies, including malignancy, particularly when clinical suspicion remains high or thoracic lymphadenopathy is present (13, 16). As interventional pulmonology increasingly adopts minimally invasive approaches such as robotic navigational bronchoscopy, awareness of these limitations remains essential (21).

This single-center retrospective study examines the characteristics of malignancy associated with organizing pneumonia (OP), but several limitations should be noted. Selection and sampling bias may exist due to the predominance of surgical specimens (Cohort 1) and inclusion of cases after non-diagnostic biopsies, which could overrepresent complex or high-suspicion cases. Variability in pathology reporting over the 13-year period introduces potential information bias and limited our ability to assess the proximity of OP to the primary tumor. Additionally, whether advances in bronchoscopy techniques, such as larger cryobiopsies, would alter the detection of concurrent malignancy in Cohort 2 remains uncertain. As Indiana University Health is a major referral center, some patient data from outside institutions were not uniformly captured despite manual chart review. Finally, staging information was unavailable for some patients due to the type of biopsy performed. Future prospective studies with standardized pathologic criteria are needed to clarify these relationships.

## Conclusion

Lung malignancy can be found concurrently with OP. The presence of OP on minimally invasive biopsy should prompt continued suspicion for cancer when clinical or radiographic features suggest malignancy.

## Data Availability

The datasets presented in this article are not readily available because requests to access the datasets should be directed to nalnasra@iu.edu.

## References

[1] Siegel RL Kratzer TB Giaquinto AN Sung H Jemal A . Cancer statistics, 2025. CA Cancer J Clin. (2025) 75:10–45. doi: 10.3322/caac.21871, PMID: 39817679 PMC11745215

[2] Hendrix W Rutten M Hendrix N van Ginneken B Schaefer-Prokop C Scholten ET . Trends in the incidence of pulmonary nodules in chest computed tomography: 10-year results from two Dutch hospitals. Eur Radiol. (2023) 33:8279–88. doi: 10.1007/s00330-023-09826-3, PMID: 37338552 PMC10598118

[3] Wahidi MM Lee S Cramer GR Cangelosi MJ . Sampling of thoracic lymph nodes and lung lesions: trends in procedural utilization. Respiration. (2023) 102:495–502. doi: 10.1159/000530741, PMID: 37290401

[4] Lentz RJ Frederick-Dyer K Planz VB Koyama T Aboudara MC Avasarala SK . Navigational bronchoscopy or transthoracic needle biopsy for lung nodules. N Engl J Med. (2025) 392:2100–12. doi: 10.1056/NEJMoa2414059, PMID: 40387025 PMC12640718

[5] Gonzalez AV Silvestri GA Korevaar DA Gesthalter YB Almeida ND Chen A . Assessment of advanced diagnostic bronchoscopy outcomes for peripheral lung lesions: A delphi consensus definition of diagnostic yield and recommendations for patient-centered study designs. An official American thoracic society/American college of chest physicians research statement. Am J Respir Crit Care Med. (2024) 209:634–46. doi: 10.1164/rccm.202401-0192ST, PMID: 38394646 PMC10945060

[6] Henderson LM Su IH Rivera MP Pak J Chen X Reuland DS . Prevalence of lung cancer screening in the US, 2022. JAMA Netw Open. (2024) 7:e243190. doi: 10.1001/jamanetworkopen.2024.3190, PMID: 38512257 PMC10958241

[7] Vieira AL Vale A Melo N Caetano Mota P Jesus JM Cunha R . Organizing pneumonia revisited: insights and uncertainties from a series of 67 patients. Sarcoidosis Vasc Diffuse Lung Dis. (2018) 35:129–38. doi: 10.36141/SVDLD.V35I2.6860, PMID: 32476892 PMC7170093

[8] Ding QL Lv D Wang BJ Zhang QL Yu YM Sun SF . Macrolide therapy in cryptogenic organizing pneumonia: A case report and literature review. Exp Ther Med. (2015) 9:829–34. doi: 10.3892/etm.2015.2183, PMID: 25667636 PMC4316910

[9] Melloni G Cremona G Bandiera A Arrigoni G Rizzo N Varagona R . Localized organizing pneumonia: report of 21 cases. Ann Thorac Surg. (2007) 83:1946–51. doi: 10.1016/j.athoracsur.2007.01.062, PMID: 17532376

[10] Radzikowska E Nowicka U Wiatr E Jakubowska L Langfort R Chabowski M . Organising pneumonia and lung cancer - case report and review of the literature. Pneumonol Alergol Pol. (2007) 75:394–7. doi: 10.5603/ARM.27961, PMID: 18080991

[11] Zhao F Yan SX Wang GF Wang J Lu PX Chen B . CT features of focal organizing pneumonia: an analysis of consecutive histopathologically confirmed 45 cases. Eur J Radiol. (2014) 83:73–8. doi: 10.1016/j.ejrad.2013.04.017, PMID: 23711424

[12] Eguchi T Takasuna K Fujiwara M Yoshida K . Coexistence of a pulmonary adenocarcinoma with a focal organizing pneumonia. Interact Cardiovasc Thorac Surg. (2011) 13:444–6. doi: 10.1510/icvts.2011.275065, PMID: 21791518

[13] Huo JP Liu C Jin BB Duan FX Mei SH Li XG . Cryptogenic organizing pneumonia masquerading as lung carcinoma: A case report and review of the literature. Exp Ther Med. (2018) 15:39–46. doi: 10.3892/etm.2017.5393, PMID: 29399056 PMC5769272

[14] Mao R Zhang L Hou J Zou Y Zhu L Chen Z . Organizing pneumonia secondary to lung cancer of unknown primary site. Respir Med Case Rep. (2019) 28:100892. doi: 10.1016/j.rmcr.2019.100892, PMID: 31312597 PMC6610690

[15] Romero S Barroso E Rodriguez-Paniagua M Aranda FI . Organizing pneumonia adjacent to lung cancer: frequency and clinico-pathologic features. Lung Cancer. (2002) 35:195–201. doi: 10.1016/S0169-5002(01)00405-6, PMID: 11804693

[16] Ichikawa T Hattori A Suzuki K Matsunaga T Takamochi K Oh S . Clinicopathological characteristics of lung cancer mimicking organizing pneumonia on computed tomography-a novel radiological entity of pulmonary Malignancy. Jpn J Clin Oncol. (2016) 46:681–6. doi: 10.1093/jjco/hyw053, PMID: 27174957

[17] Duma N Santana-Davila R Molina JR . Non-small cell lung cancer: epidemiology, screening, diagnosis, and treatment. Mayo Clin Proc. (2019) 94:1623–40. doi: 10.1016/j.mayocp.2019.01.013, PMID: 31378236

[18] Travis WD Brambilla E Nicholson AG Yatabe Y Austin JHM Beasley MB . The 2015 world health organization classification of lung tumors: impact of genetic, clinical and radiologic advances since the 2004 classification. J Thorac Oncol. (2015) 10:1243–60. doi: 10.1097/JTO.0000000000000630, PMID: 26291008

[19] King TE Jr. Lee JS . Cryptogenic organizing pneumonia. N Engl J Med. (2022) 386:1058–69. doi: 10.1056/NEJMra2116777, PMID: 35294814

[20] Romero S Barroso E Rodriguez-Paniagua M Aranda FI . Organizing pneumonia adjacent to lung cancer: frequency and clinico-pathologic features. Lung Cancer. (2002) 35:195–201. doi: 10.1016/S0169-5002(01)00405-6, PMID: 11804693

[21] Fernandez-Bussy S Funes-Ferrada R Yu Lee-Mateus A Vaca-Cartagena BF Barrios-Ruiz A Valdes-Camacho S . Transforming lung cancer diagnosis: the role of robotic-assisted bronchoscopy in early detection and staging. Lung Cancer. (2025) 206:108646. doi: 10.1016/j.lungcan.2025.108646, PMID: 40602203

